# Pattern of Pseudoexfoliation Deposits on the Lens and Their Clinical Correlation- Clinical Study and Review of Literature

**DOI:** 10.1371/journal.pone.0113329

**Published:** 2014-12-05

**Authors:** Aparna Rao, Debananda Padhy

**Affiliations:** Glaucoma Service, LV Prasad Eye Institute, Patia, Bhubaneswar, Odisha, India; The University of Tennessee Health Science Center, United States of America

## Abstract

**Purpose:**

To study the clinical correlates of pattern of deposits over the lens in patients with pseudoexfoliation syndrome (PXF) or pseudoexfoliation glaucoma.

**Methods:**

This retrospective observational study screened 346 patients with PXF seen in glaucoma clinic of a tertiary hospital from 2011–2013. Details like pattern of deposits, location on the lens surface and pupillary abnormalities in slit lamp photographs and their correlation with clinical and demographic variables, were analysed.

**Results:**

A total of 84 eyes of 42 patients with bilateral PXF were included for the study. Glaucoma was seen in 30 eyes with baseline IOP of 24+3.8 mm Hg. Comparing the type of deposits, namely classical (n = 39 eyes), radial pigmentary (RP) form (n = 39 eyes) and combined classical and radial pigmentary (CR) forms (n = 6 eyes) of deposits, pupillary ruff atrophy was common in all forms while poor dilatation was rare in the RP type (n = 5 vs n = 25 in classical forms, p<0.001). Mean deviation (MD) was worse in the classical and CR form as compared to RP type with the latter presenting much earlier, 43±3.2 years vs 48±4.1 years in CR and 56±5.7 years in classical form, p<0.001. The baseline IOP in the RP group (18±2.3 mm Hg) was significantly lower than the other two forms (CR 20±3.2 mm Hg, classical 28±2.3 mm Hg), p<0.001, with only 2 eyes on anti-glaucoma drugs at presentation.

**Conclusion:**

Pattern of exfoliation deposits may indicate the stage and severity of the disease process in evolution with the RP representing an earlier/less severe form of pseudoexfoliation syndrome.

## Introduction

Pseudoexfoliation syndrome (PXF) represents a unique age related disease characterised by deposition of extracellular fibrillar material over ocular tissues. [1], [2] In the eye, deposits are seen more commonly in the anterior segment, mainly the anterior lens capsule. [3]–[6] The exact origin of this exfoliative material composed of components of the fibrillar system, remains a mystery. This entity is a systemic disorder with deposits seen in many ocular tissues and other tissues in the body like meninges and liver. [4], [7] The other ocular structure commonly affected is the iris, involvement of which can be evidenced by poor dilatation, transillumination defects and exfoliative deposits at the margin of iris vessels demonstrated on electron microscopy. The deposits on the lens capsule are classically seen as a peripheral ring of dandruff like material, an intervening clear zone and a central zone. The central disk is reported to be absent in 20%–60% of cases. [4], [5] Ritch et al has observed that a homogeneous ground-glass or matte appearance of the lens surface in one eye compared to the other may represent a very early (precapsular) stage. [5] In a perhaps slightly later (pregranular) stage, authors postulated that a ring of about 80 faint, radial, non-granular striae may be seen on the mid-third of the anterior capsule behind the iris. Ultra structurally, the precapsular layer has been shown to consist of microfibrils, but not mature exfoliation fibres. Ritch et al has elucidated different phenotypes of the peripheral ring as also features of early disease (precapsular and pregranular). Yet, they have not compared clinical features with different phenotypes of the disease process.

While both bilateral and unilateral cases are common, several clinical differences including older age, marked pigmentation and higher baseline intraocular pressure (IOP) in the former have been reported. [6], [7] Phenotypic differences in disc and Spectral domain optical coherence tomography features in bilateral and unilateral cases have also been reported. [8] It is however unknown if different patterns of exfoliation deposits have any relation to severity or clinical associations in this distinct entity. Though this disease is now recognised as a separate entity with worse prognosis compared to primary open or closed angle glaucoma, elucidation of its different phenotypes is far from complete [8]–[10]. While the severity of pseudoexfoliation or extent of deposits has not been found to correlate with intraocular pressure, trabecular meshwork pigmentation or extent of glaucomatous damage, [9] the clinical correlates of phenotypically different types of deposits over the lens has not been evaluated earlier. Such a study would possibly provide a clue to the factors correlating with extent of glaucoma or perhaps the origin of the deposits.

## Methods

This retrospective study included patients with PXF screened in the outpatient department for a study evaluating long term course of different types of glaucoma, which was approved by the institutional review board of LV Prasad Eye institute, Odisha, India and adhered to the tenets of declaration of Helsinki. As protocol, a written informed consent is taken for all patients attending our clinic for investigations and examination. Details retrieved included slit lamp evaluation with slit lamp photographs, intraocular pressure (IOP) by Goldman applanation tonometry, 4 mirror gonioscopy for assessing angle and extent of pigmentation of the trabecular meshwork (TM) graded as mild, moderate or severe, +90D fundus biomicroscopy and Humphrey visual fields (Carl Zeiss Inc, Dublin, CA, USA, 24-2 SITA standard program).

Inclusion criteria for pseudoexfoliation syndrome included those with evident classical dandruff or flaky exfoliation deposits on the pupil, lens or other ocular structures, radial pigment over the lens surface with or without raised intraocular pressure. Only clinically bilateral eyes were included for analysis (since clinically unilateral cases could have been bilaterally asymmetric cases which could not be verified in a retrospective review of records). Glaucoma was defined as those with glaucomatous optic neuropathy evidenced by cupping, rim thinning, notch or retinal nerve fibre layer defects with corresponding visual field defects. Patients with uveitis, neovascular glaucoma, aphakia or pseudophakia or past laser procedures were excluded. Patients with associated findings like uveitis, previous laser or surgery, trauma or posterior segment pathology were excluded.

Visual field defects were classified as glaucomatous if glaucoma hemifield test outside normal limits or pattern standard deviation with probability <5%, which were reproducible over three baseline fields.

Slit lamp photos were captured by the same observer (DP) in low and high magnification after full dilatation with tropicamide 0.5% and phenylephrine 5% using Eye Cap system (Haag Street International, Koeniz Switzerland) while video options allowed for continuous video capturing of the type of deposits. Details like pattern of deposits, location on the lens surface (central or peripheral), presence (or absence) of deposits over pupillary ruff, pupillary abnormalities like ruff atrophy were noted. The deposits were characterised further by noting presence or absence of curling of the exfoliative deposits, presence of empty spaces within the deposits and pattern of deposits. The extent of pupillary dilatation, clinical/demographic variables and pattern of deposits were also compared between two eyes of bilateral cases.

All statistical analysis was done using Stata (Stata Corp, Version 12, CA, USA) with p<0.05 defined as statistically significant. Since each eye in bilateral pseudoexfoliation is reported to be in different stages of evolution and severity [1], [3], each eye of patient was considered as a separate entity for all analysis and comparisons to evaluate differences in pattern of deposits in each eye with the clinical features in that particular eye. Since the two eyes were not considered to be correlated with respect to severity and pattern of deposits, clustered statistical analysis, which smoothens out any correlation between two eyes of the same patient, was not applicable and therefore not performed for analysis in this study. For detecting a difference in MD or IOP more than 5dB or 5 mm Hg between groups with 90% power and significance set at p<0.05, the sample size required for such a study was calculated to be 5 and 3 respectively.

Frequency of pattern of exfoliative deposits was evaluated using chi-square statistics while continuous variables between groups were compared using one way ANOVA statistics. Correlation between pattern of deposits, field defects, clinical and demographic variables including IOP and disc damage was analysed using logistic regression.

## Results

Of 346 patients with pseudoexfoliation screened from 2011–2013, a total of 123 patients with documented clinical photographs by the same technician (DP) fulfilling inclusion criteria were included for the study. Of those excluded, 144 had history of trauma, neovascular glaucoma or posterior segment involvement (retinal vein occlusive disease). A total of 112 patients were excluded because of aphakic/pseudophakic status in one or both eyes while 72 patients with no reliable visual field data and 8 with no fundus view due to significant cataract were also excluded. Of 122 selected, 81 with poor quality photographs (where pattern of deposits was not clear or poorly focussed images) or unco-operative patients were excluded. A total of 84 eyes of 42 patients were included for the study which comprised of 34 males.

All patients had bilateral disease with a mean age of 58±6.5 years. Glaucoma was present in 30 eyes on a mean of 2+1.2 medicines at presentation and a baseline IOP of 24+3.8 mm Hg (**Table 1**).

**Table 1:** Demographic and clinical characteristics of patients with bilateral pseudoexfoliation syndrome.

Variables	Mean±SD
Age (years)	58±6.5
Males∶females	34∶9
PXF∶PXG	36∶30
Untreated baseline IOP (mm Hg)	24±4.8
MD (dB)	−11±10.4
No of medicines	2±1.2

IOP-Intraocular pressure; PXF-Pseudoexfoliation syndrome; PXG-Pseudoexfoliation glaucoma.

### A-Pattern of exfoliation deposits

**Figure 1:**
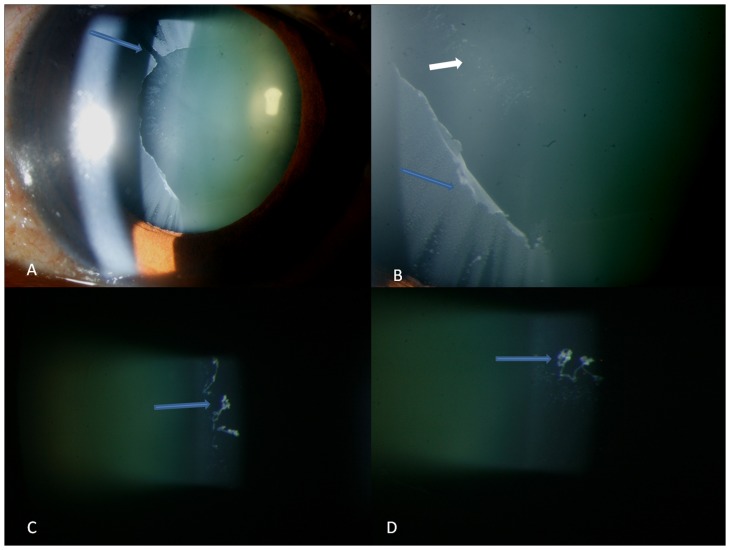
Figures showing additional features of classical pseudoexfoliation like slit shaped empty spaces (arrow in top left), anterior curling of the exfoliation sheet (blue arrow, top right) with pin-point deposits in a wave like pattern anterior to the sheet (small white arrow) which on higher magnification shows globular protrusions at the end of a stalk arising from the edge of the wave (arrows in bottom left and right).

Analysing pattern of deposits, a total of 39 eyes had classical deposits (**Figure 1** **,** **2** **&** **Figure S1**) while 39 had radial pigments (henceforth termed RP form, **Figure 3** **&** **Figure S2**). Six eyes of 5 patients presented with combined features of classic and pigmentary deposits (henceforth termed CR form, **Figure 4**). There was no difference in the extent of TM pigmentation on gonioscopy between the different forms of the disease with all eyes displaying severe pigmentation at the angle.

**Figure 2:**
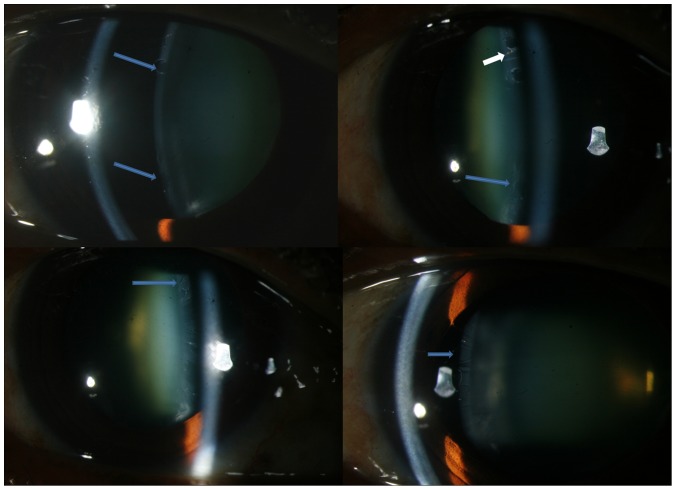
Slit lamp photograph showing atypical features of classical pseudoexfoliation like circularly shaped empty spaces (Top left &right, bottom left and right) which are seen extending from the periphery to the centre interspersed with pigment deposits (white arrow in top right).

**Figure 3:**
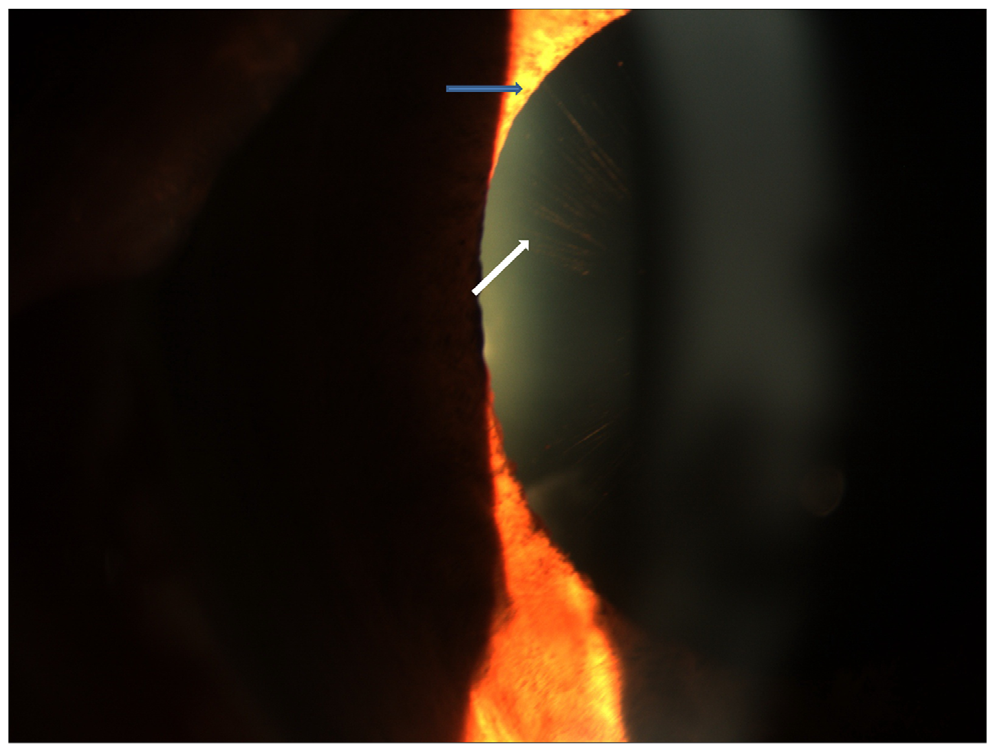
Slit lamp photograph showing radial pigmentary pattern of deposits on the anterior lens surface (white arrow) with adjacent pupillary ruff atrophy (blue arrow).

**Figure 4:**
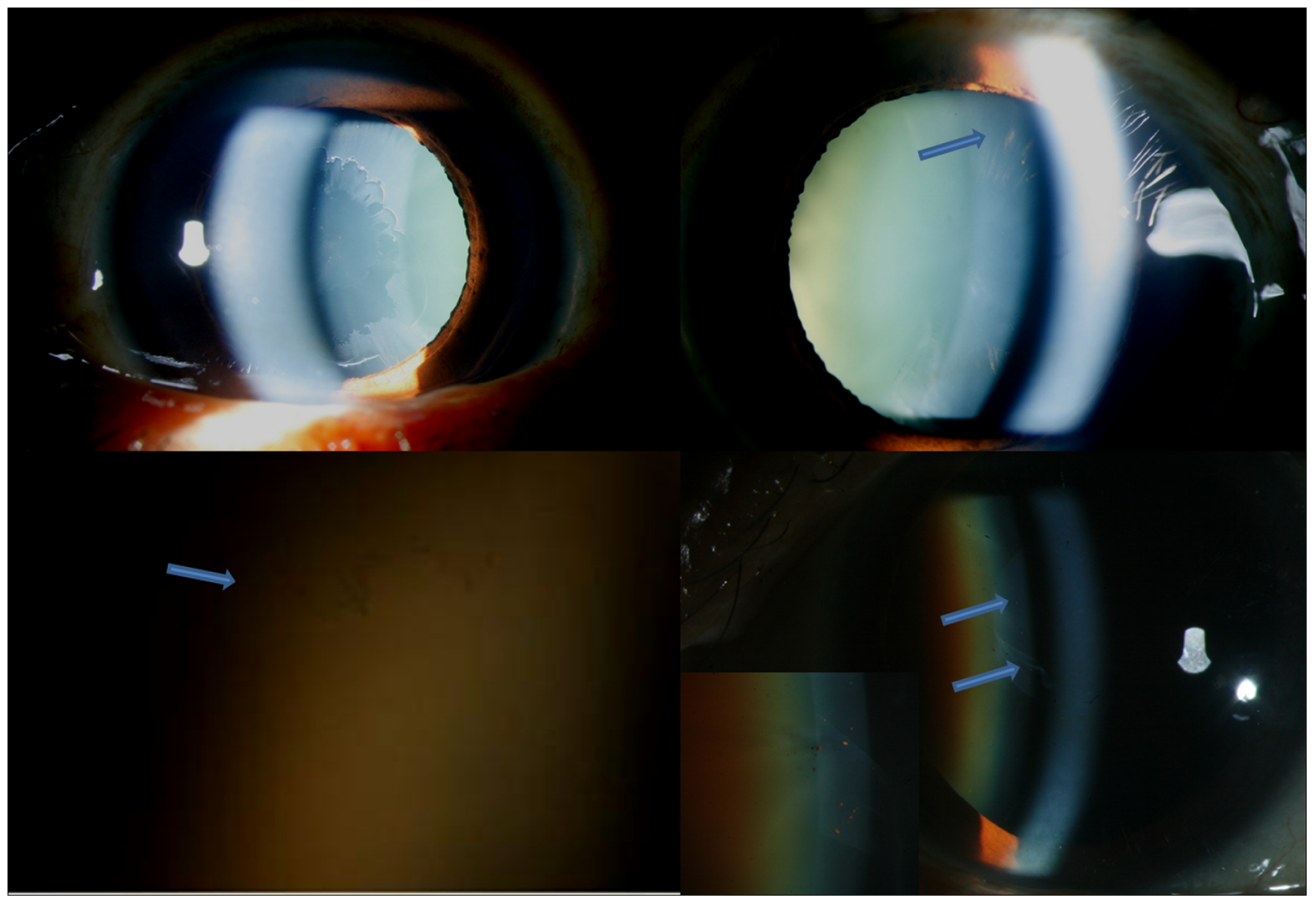
Slit lamp photograph showing right eye (Top left) classical peripheral pseudoexfolative ring and left eye (Top right) showing radial pigments pattern of deposits. Bottom left shows slit lamp photograph in high magnification showing coalescence of peripheral radial pigments resembling early classical peripheral ring (arrow). Bottom right shows slit lamp photograph showing central classical pseudoexfoliation ring with anterior curling of the sheet (Blue arrows) with inset showing interspersed radial pigment deposits.

Of 39 eyes with classical or RP form of lens deposits, 30 (of 15 patients) and 28 (of 14 patients) eyes were bilateral pure classical or RP forms of pseudoexfoliation respectively. Eight patients (16 eyes) had classical form in one eye and RP form in the contralateral eye. Of 6 eyes of 5 patients with CR form of deposits, 2 eyes of the same patient had pure CR forms in both eyes while contralateral eye of 4 patients with combined form had classical (n = 1) or RP form (n = 3).

#### i) Classical form of deposits

The deposits were distributed in the periphery in all 39 classical cases while 17 eyes had central ring of deposits also in addition. Of 39 with classical deposits, 17 eyes had empty spaces, rounded empty spaces were found in 2 eyes (**Figure 2**) while others had slit shaped empty defects (**Figure 1** and Figure S1). The eyes with round empty spaces were atypical in having diffuse distribution of peripheral PXF deposits extending to the pupillary centre with no central zone seen (**Figure 2**). Curling of the deposits axially over the lens capsule was seen in 29 eyes with one eye showing severe curling of the membrane simulating ruptured anterior capsular membrane with no corneal touch (**Figure** 1). Such findings were however consciously absent in the 39 eyes with RP or CR form of deposits. There was evidence of pin-point exfoliative deposits anterior to the curled up exfoliative membrane which on high magnification revealed deposits to be distributed in a wave like pattern. There was a stalk of thin white deposits arising from the origin of the wave which bore globular deposits (**Figure 1**) at its end towards the edge of the wave or at the “bank”. Comparing the phenotype with clinical features, 26 eyes with glaucoma requiring medical treatment had poorly dilating pupils (n = 25) and curling of the anterior capsular membrane (n = 26) were associated with higher IOP (18±5.4 mm Hg) and worse mean deviation (−19±3.8 dB) as compared to eyes with classical PXF pattern of deposits without glaucoma (−14±3.2 and −10±3.7, respectively), p<0.001.

#### ii) Radial pigmentary form

The RP form had peripherally arranged fine radial pigmentary lines on the anterior lens surface extending from the periphery to the centre and sparing the pupillary area (**Figure 3**). There was intervening clear space between the pigmentary lines with occasional rounded pin-point pigment collections (**Figure 3** **and** **Figure S2**) interspersed between the radial lines with no curling of the capsule seen in any eye.

Comparing phenotypes with clinical features, 2 eyes with lens induced glaucoma was not associated with any clinical parameter like ruff atrophy or poor dilatation.

Three eyes showed conglomeration of the peripheral lines with no clear space (**Figure 4** **& Video S1**) resembling early classical form of pseudoexfoliation deposits.

#### iii) Combined form

Combined (CR) form of pseudoexfoliation showed features of classical peripheral or central ring with radial pigmentary pattern in the same eye (distinct from the scattered pigment deposition seen in pseudoexfoliation syndrome, **Figure 4**).

The power for this study using the differences in clinical parameters like IOP or MD in each group was 98 and 99% respectively.

### B-Pupillary abnormalities

Pupillary ruff atrophy was seen in 79 eyes (**Figure 3**) while pupillary deposits were seen in 23 eyes. Poor dilatation was seen in 30 eyes (5 RP and 25 classical form) which was associated with presence of deposits on pupil and presence of curling of lens deposits in the classical form, p<0.001 each. Pupillary deposits were more common in the classical form of the disease (n = 18) than the other two forms (n = 1 each), p<0.001. Poor dilatation was however not associated with extent of lens deposits or ruff atrophy, p = 0.6 and 0.5, respectively.

### C-Forms of exfoliation deposits and clinical features

Comparing phenotypes with clinical features, 2 eyes with glaucoma in eyes with RP form of deposits were not associated with any clinical parameter like ruff atrophy or poor dilatation. Two eyes with CR form had poor dilatation with one eye showing curling of the capsular membrane axially, though this was not statistically significant.

Comparing the type of deposits, namely classical, RP form and CR forms of lens deposits, pupillary ruff atrophy was common in all forms while poor dilatation was rare in the RP type (n = 5 vs n = 25 in classical forms, p<0.001). Mean deviation (MD) was worse in the classical and CR form (**Table 2**) as compared to RP type with the latter presenting much earlier, 43±3.2 years vs 48±4.1 years in CR and 56±5.7 years in classical form, p<0.001. The baseline IOP in the RP group (18±2.3 mm Hg) was significantly lower than the other two forms (CR 20±3.2 mm Hg, classical 28±2.3 mm Hg), p<0.001, with only 2 eyes on anti-glaucoma drugs at presentation. The cause for raised IOP was subluxated cataract in both eyes with secondary angle closure.

**Table 2:** Comparison of clinical variables in different forms of pseudoexfoliation lens deposits.

Variables	Classical	RP	CR	
Age (years)	56±5.7 years	43±3.2 years	48±4.1 years	<0.001
PXG	26	2	2	<0.001
Untreated baseline IOP	28±2.3	18 ±2.3	20±3.2	<0.001
MD	−12±6.2	−4±2.3	−7±2.8	<0.001
No of eyes on medicines	26	2	2	0.005
Poor dilatation	25	5	0	<0.001
Ruff atrophy	30	26	23	0.5
Pupillary deposits	18	1	1	<0.001

IOP-Intraocular pressure; PXG-Pseudoexfoliation glaucoma; RP-Radial pigmentary form of exfoliation deposits; CR-Combined classical and radial pigmentary pattern of exfoliation deposits.

Analysing difference between two eyes of the same patient, 8 had either classical pseudoexfoliation signs in one eye while contralateral eye had isolated pigmentary deposits. The eye with classical signs had higher baseline IOP, p<0.001, and worse mean deviation, p = 0.01. Of 8 patients, 6 eyes with classical signs were on medical treatment (mean of 1±2.1 drugs) for raised IOP while 2 eyes with RP form were on medical treatment, p = 0.02.

Comparing the features of CR form in 6 eyes of 5 patients, eyes with radial pigment in the contralateral eye (n = 3) had lower baseline IOP (16±2.1 mm Hg) and better mean deviation (−3±1.2 dB), than the eye with contralateral classical form of deposits (n = 1, 28 mm Hg and −21 dB), p<0.001 of each.

## Discussion

Pseudoexfoliation syndrome is a complex matrix of disease which transits slowly from a milder form to more severe forms with eventual glaucomatous optic neuropathy. [4]–[7] The glaucoma that results in PXF is difficult to control with a worse prognosis and has a faster rate of progression with frequent vascular ischemic episodes. [4], [5], [10], [11] These features of PXF have merited classification of PXF into a separate entity and exclusion from primary adult forms of glaucoma, namely primary open angle glaucoma, POAG, and primary angle closure glaucoma, PACG. Yet, characterisation of the clinical findings of different phenotypes in different stages has not been evaluated so far. This study found eyes with radial pigmentary type of lens deposits to present earlier, have lower baseline IOP and better MD at presentation, indicating an early form of pseudoexfoliation disease process. While poor dilatation was not universal in all cases of PXF as believed, classical form of the disease with poor dilatation and curling of the exfoliative deposits was associated with worse mean deviation and higher baseline IOP at presentation. This however was not observed in the other two forms of the disease in this study.

Bartholomew et al has described a pregranular form consisting of 80 faint radial pigmentary lines in early PXF. [11] The author has clearly demonstrated stages of the pigmentary deposits with 5 eyes in this study having granular PXF ion one eye and pre-granular in the other eye. This form was most prevalent in the 30–39 year old aged Bantu Africans which dropped to 1 at the age of 70 years. There was a corresponding increase in the granular form of PXF with age. Though pre-clinical forms have been described, [12]–[14] clinical correlation of this stage of disease has not been done earlier. The pigment lines have been reported to consist of microfibrils rather than exfoliation fibres. [4], [15] Ritch et al have described a pre-capsular form with ground glass appearance of the lens capsule representing early PXF disease. [4] Yet, none of the earlier studies have evaluated the form of PXF deposits to the clinical features seen or evaluated reasons for different patterns.

There could be several plausible reasons for the radial pattern of pigmentation seen. Several authors have identified microneovascualrisation, hypoperfusion and anastomotic tufts on iris flourescein angiography (IFA). [16]–[21] One study evaluating IFA pattern in PXF observed that patients clinically affected only monolaterally by PXF show microvascular changes, which are similar in both eyes. [20] The authors concluded that glaucoma represents a more advanced stage of the disease with more pronounced alterations, even though no unique microvascular pattern could be identified by iris angiography. Our study showing different patterns of clinically manifest PXF deposits may represent different stages of disease evolution. Parodi et al observed that eyes affected by capsular glaucoma showed signs of microneovascularisation (marked stromal tufts and marked plexi), and anastomotic vessels (peripheral loop, lesser circle and oblique vessels). [20] The microvascular abnormalities at a microscopic level may be responsible for the different phenotypes, (empty spaces of different shapes in classical PXF corresponding to vascular loops or occasional pigment rounding interspersed between radial pigments corresponding to oblique vessels in this study) representing areas of anastomic tufts or different patterns of neovascularisation at different locations giving rise to the diverse pattern of deposits in this entity. These would therefore suggest that the source of the deposits in the eye to be the iris blood vessels (explaining why this is a systemic disease with vascular ischemic episodes) while different ocular features or pattern of deposits are due to different epigenetic influences.

Pigmentary deposits on the lens are very common in many ocular diseases like uveitis, trauma, pigment dispersion or angle closure disease. Yet, none of these cases were associated with retrocorneal pigmentation or other signs of uveitis, which was carefully screened and excluded. A radial pattern of deposits as seen in this disease signifies deposits arising from the iris vessels due to breakdown of blood aqueous barrier or may indicate chronic rubbing of the iris against an ageing lens. The pattern of these deposits is uniquely seen as peripheral, fine and radially oriented [11] (corresponding to radial vessel pattern of the iris) which differ phenotypically from scattered circular or localised pigmentary deposits seen in mid-periphery or blotchy deposits seen in uveitis or trauma. In later stages, the peripheral lines were observed to coalesce indicating that the RP may denote an earlier stage of the disease process along with pupillary ruff atrophy. This now points to the possibility that the classical PXF deposit so commonly seen in the anterior segment arises from chronic rubbing of two surfaces with exudation from vascular structures in close apposition between the two surfaces. This theory may need to be proved in other structures in the body like meninges, liver or others where friction between the organ and its external covering containing vessels may be responsible for deposits seen on these tissues. [4], [7] From a clinical perspective, early lens removal in the earlier stages may theoretically prevent further progression of the disease. [22] Some of the eyes with classical form showed a wave like pattern of deposits anterior to the curled up classical exfoliation membrane with globular deposits atop a stalk, indicating that these deposits were washed off in a wave form resultant to iris movement (or rather pupillary movement under physiologic conditions) over the lens. The globular deposits at the “bank” would therefore represent deposits which were washed to the “shore” (periphery to centre) by iris movement. This point towards the origin of these deposits from the iris (close to the iris vessels) rather the lens.

This study observed poor dilatation and axial curling of the anterior capsular membrane to be significantly associated with glaucoma in the classical form only. Poor dilatation is known to be associated in PXF eyes even without the use of miotics. [4], [7], [23]–[26] In unilateral or bilateral cases, the involved eye has been noted to smaller in the undilated state. [3]–[5] One study evaluating histological changes in the dilator muscle showed disorganised and degenerative muscle fibres. [25] If the RP or CR forms represent earlier forms of the disease process in evolution, it is logical that degenerative changes typical of chronic disease would be absent, as observed in this study. These observations add evidence that the other two forms represent early disease which therefore was not associated with classically described associated features. Longitudinal studies and possibly molecular markers in the aqueous indicating early oxidative stress would indicate possible prognostic factors for eyes that are likely to develop glaucoma after detection of the RP form. Further, poor dilatation or identifying the axial pattern of deposits in eyes with classical form may indicate those likely to develop glaucoma.

Thirty of 39 eyes with classical form, 2 of 6 eyes with combined form and 2 with RP form had raised IOP with disc changes at presentation. Causes of raised IOP in PXF are postulated to be blockage of the TM by PXF material or by extracellular matrix (ECM) remodelling leading to TM or lamina cribrosa structural alterations. [4]–[7], [9], [27], [28] It has been reported that the severity of the lens deposits does not correlate with extent of glaucomatous damage or trabecular pigmentation. [9], [27] While the stage at which ECM remodelling starts in the disease process is unknown, it may be possible that chronic rubbing of the iris with the lens leads to characteristic changes in the adjacent iris blood vessels triggering protein precipitation onto the lens surface. Later stages with chronic rubbing may cause larger molecular weight proteins precipitation out due to loss of chaperone effect of major proteins like clusterin. This raises the possibility that molecular changes leading to TM damage leading to raised IOP may rise irrespective of extent, stage or pattern of exfoliation deposits, which therefore points towards non-mechanical causes of trabecular damage in this disease. Epigenetic influences like high temperature may influence the extent of breakdown of blood aqueous barrier and precipitation of proteins. [29] This may possibly explain higher prevalence of this entity in some populations and lack of gene associations in different ethnic populations. Chronicity of iris rubbing over the lens and the lens thickness may perhaps be the only determinants of trabecular dysfunction rather than extent of deposits or TM pigmentation. While this might be a conjecture at this time point, sampling the level of protein expression in the anterior chamber in these different forms of PXF would help confirm this theory, which also might provide us a clue to the mechanism of production of PXF deposits and the disease process.

There are obvious differences in bilateral and unilateral pseudoexfoliation syndrome with the former presenting in older patients. Bilateral cases are known to have worser mean deviation, higher IOP or thinner RNFL thickness compared to unilateral cases.^3,8^ This entity is considered to be bilaterally asymmetric disease with each eye in different stages of evolution [1], [3], [8]. The severity of deposits or clinical features in bilateral cases are known to vary between the two eyes [3], [8]. In this study, we included bilateral cases to evaluate differences in pattern of deposits (which serves as surrogate for local eye specific factors) versus the clinical features seen in that eye negating the effect of systemic factors which are presumed to be acting equally in both eyes. The study objective of correlating the pattern of deposits with the clinical feature in each eye in bilateral cases would therefore have been confounded if we had a mixed cohort including unilateral pseudoexfoliation syndrome which have been reported to become bilateral over time in several earlier studies. In one of our earlier study, we found ruff atrophy indicating retinal nerve fibre layer loss in the contralateral normal eye of clinically unilateral cases. [8] This study found significant differences in pattern of deposits in both eyes of bilateral PXF, as reported earlier [1], [3]; Yet, clinical correlates in these eyes were also different as seen in this study, which has not been evaluated earlier. This study also found ruff atrophy in all 3 forms of PXF suggesting that chronic rubbing of the iris over the lens surface causing ruff atrophy can indicate early PXF disease before clinically manifest flaky deposits. Therefore, this may be a sign of early disease process before the clinically evident form. This would have to be corroborated with electron microscopy of the eyes or iris flourescein angiography, which was however not studied in the current study. While pathogenesis of ruff atrophy PXF is unknown, it may indicate mechanical pigment release by rubbing or subclinical ischemic changes resultant to microvascular anomalies reported in PXF. Ruff atrophy therefore possibly portends early breakdown in blood aqueous barrier in the eyes and therefore would also indicate early ischemic process in the clinically unmanifest stage of PXF.

The evolution and pattern of exfoliation deposits may be different in different stages, as is evident in this study. The composition of the deposits is also expected to vary with the stage of the disease. Essentially this may suggest that larger agglomerates of proteins precipitate out of blood vessels in these eyes with altered blood aqueous barrier which may mechanically block the trabecular meshwork in the later stages causing refractory glaucoma. Vessel hyalinization due to blockage of vessel lumen by large intravascular aggregates of large molecular weight proteins may also cause ischemic damage in earlier or later stages. These vascular changes in evolution may be responsible for different phenotypic presentation in PXF and lack of correlation of different phenotypes with extent of IOP rise or TM damage. Further studies evaluating the vascular anomalies, protein expression and epigenetic influences with the clinical findings are required to answer key questions for this mysterious entity.

Based on this study and our observations, we suggest that PXF stages may be seen in three clinical forms-

a) RP form (earlier described pregranular form) –Here the altered blood aqueous barrier would be disturbed which would cause pigment deposition by iris friction with the anterior lens surface evident as radial pigment with normal IOP. The only clinical feature pointing to PXF in these eyes would be pupillary ruff atrophy. While glaucoma is not very common, secondary lens induced glaucoma may be seen at this stage.

b) RP with classical or combined CR form with early coalescence of deposits into classical form of peripheral ring-in thus stage, the exudation of low molecular weight proteins caused by loss of chaperone effect of certain proteins and chronic rubbing cause alteration in the pattern and type of exfoliative deposits with slow and gradual trabecular dysfunction by ECM remodelling and/or mechanical damage. These features may be bilaterally asymmetric.

c) Classical PXF with iris stromal hyalinisation due to precipitation of large molecular weight proteins and severe hyalinisation of iris vessels and trabecular damage evidenced by raised IOP and optic nerve damage. In these eyes, poor dilatation and curling of the anterior capsular membrane may indicate early glaucoma.

Trabecular damage may therefore start at any stage and poor dilatation may be an indirect indicator of ischemic changes and therefore early breakdown of blood-aqueous barrier and therefore subclinical TM damage. From a clinical perspective, lens removal in earlier forms, say RP form may therefore prevent further rubbing with lens surface and therefore prevent constant precipitation of proteins and progressive damage. Clinical staging of the disease would help in appropriate management decisions for preventing progression to the later stages.

We did not involve lens status/thickness and extent of cataract in each stage since that could have given parallel changes in lens protein changes and possibly explain denser cataract in certain forms of the disease. We did not perform iris angiography or other investigations to confirm early breakdown of blood aqueous barrier. While the results hold true for our population, similar studies on other ethnic populations globally would help characterise the phenotypic feature more appropriately in different stages of the disease. Longitudinal follow up are being done on eyes with pigmentary deposits which is expected to shed light on the evolution of the deposits with time with a direct correlation with appearance of clinically manifest glaucoma.

## Supporting Information

Figure S1 **Slit lamp photograph showing classical peripheral exfoliation ring on diffuse (Top left, arrows) and retroillumination (Top right, arrows pointing to slit shaped empty spaces between), isolated central ring (bottom Left, black arrow) and classical combination (bottom right) of peripheral (white arrow) and central ring (black arrows).**
(TIF)

Figure S2 **Slit lamp photograph of eyes (Top left and right, Bottom left and right) with radial pigmentary type of pseudoexfoliation deposits.**
(TIF)

Video S1 **Slit lamp videography showing radial pigmentary deposits in an eye with early coalescence in the periphery (arrows).**
(MPG)
