# Supplementary figures and images for: Pattern of Pseudoexfoliation Deposits on the Lens and Their Clinical Correlation- Clinical Study and Review of Literature
Source: PLoS One. 2014 Dec 5;9(12):e113329. doi: 10.1371/journal.pone.0113329 (PMC4257528; doi:10.1371/journal.pone.0113329)

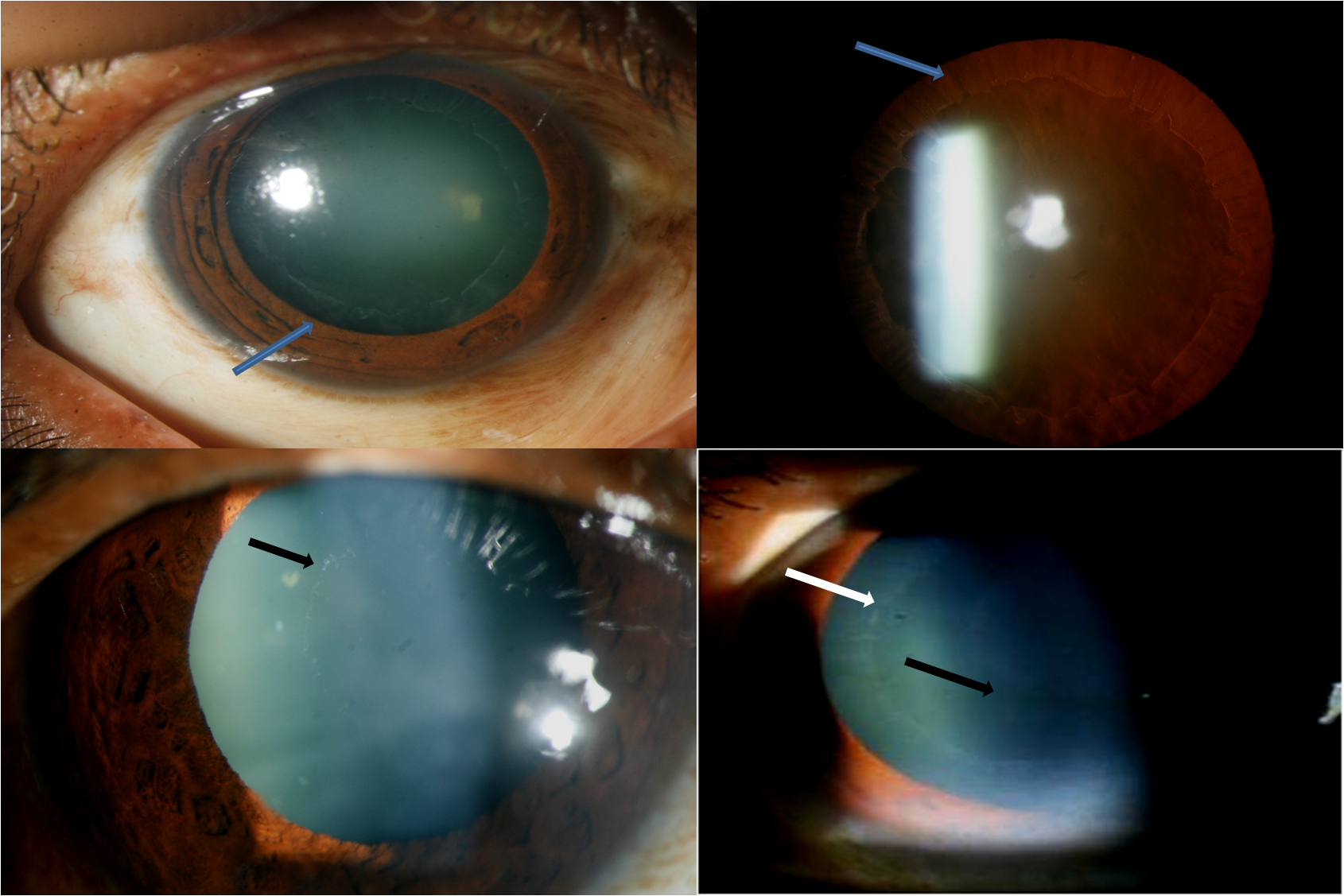

Supplement: Figure S1 — Slit lamp photograph showing classical peripheral exfoliation ring on diffuse (Top left, arrows) and retroillumination (Top right, arrows pointing to slit shaped empty spaces between), isolated central ring (bottom Left, black arrow) and classical combination (bottom right) of peripheral (white arrow) and central ring (black arrows). (TIF) [file pone.0113329.s001.tif]

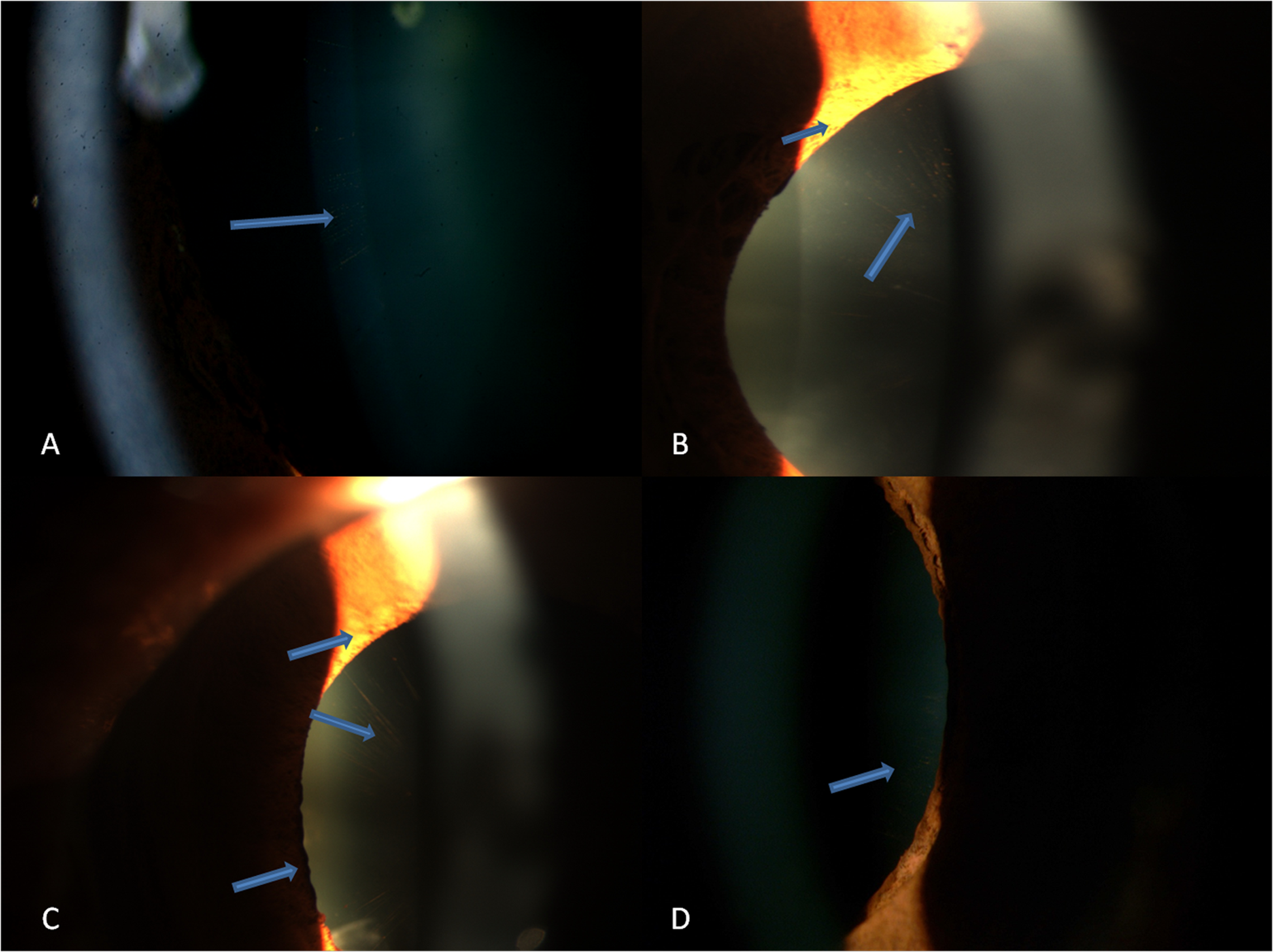

Supplement: Figure S2 — Slit lamp photograph of eyes (Top left and right, Bottom left and right) with radial pigmentary type of pseudoexfoliation deposits. (TIF) [file pone.0113329.s002.tif]
